# Erosive Pustular Dermatosis of the Scalp Induced by Imiquimod

**DOI:** 10.1155/2012/828749

**Published:** 2012-12-05

**Authors:** Maria Teresa Corradin, Marina Forcione, Erika Giulioni, Renzo Fiorentino, Anna Ferrazzi, Mauro Alaibac

**Affiliations:** ^1^Unit of Dermatology, Pordenone Hospital, 33170 Pordenone, Italy; ^2^Unit of Dermatology, University of Padua, 35128 Padua, Italy

## Abstract

Erosive pustular dermatosis of the scalp (EPDS) is a rare condition characterized by sterile pustules, erosions, and crusted lesions on the scalp of elderly patients. This inflammatory disorder has an unknown origin and it could develop into areas of alopecia that tend to be atrophic. An 84-year-old Caucasian man presented with a several months history of painful erythematous erosions and crusts on his scalp. The lesions appeared after treatment with imiquimod cream for actinic keratoses. Previous therapies included topical antibiotics and topical steroids. Physical examination revealed the presence of extensive erosions and crusts on the scalp, with minute pustules on the sides. The clinical features and the medical history led us to the diagnosis of EPDS. Treatment with systemic steroid was administered with improvement observed after ten days. The clinical manifestations of EPDS completely resolved after 2 months, without clinical relapses.

## 1. Introduction

Erosive pustular dermatosis of the scalp (EPDS) is a rare entity of unknown origin mainly affecting elderly people. It is often triggered by local trauma and is characterised by chronic pustules of the scalp evolving toward scarring alopecia. The main dermatological manifestations of EPDS include erythematous plaques, erosions, sterile pustules, and crusts [1]. Although EPDS has an unknown etiology, several predisposing factors have been reported, notably trauma, skin grafting, and prolonged exposure to UV light of a bald scalp [2]. EPDS typically develops in aged or sun-damaged scalp skin with alopecia and/or multiple actinic keratosis [1]. Skin atrophy seems to be the main causal factor of EPDS development. While prolonged exposure to UV rays and baldness are considered the most important predisposing factors, the main precipitating factor of EPDS seems to be skin trauma [3]. The lesions have been documented to follow trauma of the scalp and tend to be chronic, progressive, and difficult to treat [4]. Local trauma is generally implicated, notably cryotherapy, radiation therapy, laser therapy, and physical and chemical insults. Treatment with topical drugs are reported to be possible precipitating factor [3]. EPDS diagnosis is difficult due to the lack of specific histological features [5, 6]. The clinical differential diagnosis of EPDS includes bacterial or fungal infection, pemphigus, squamous cell carcinoma, and dermatitis artefacta [6].

Here, we report a patient presenting with EPDS following topical treatment with imiquimod, used for multiple actinic keratosis.

## 2. Case Report

**Figure 1:**
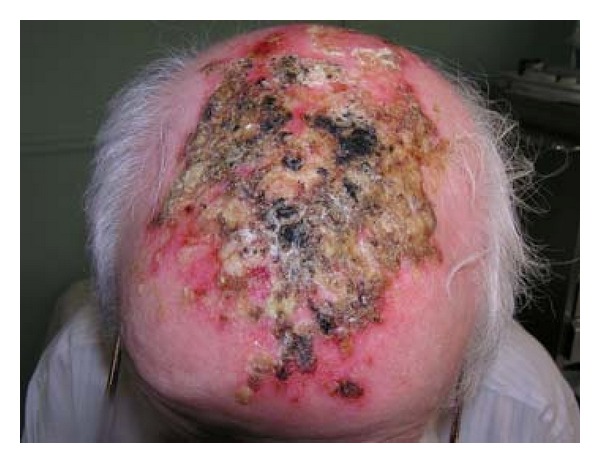
Erythematous patches and crusting developed a few weeks after topical treatment with imiquimod cream for actinic keratosis and lasted several months without spontaneous resolution.

**Figure 2:**
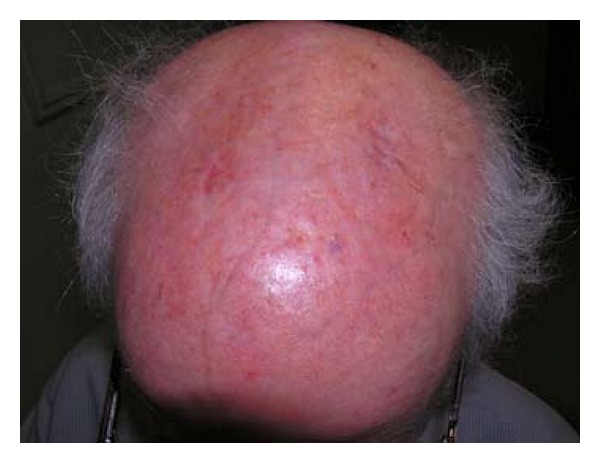
Complete resolution after 2 months of oral steroid treatment.

A 84-years-old Caucasian man presented in our unit with a chronic scalp eruption characterized by erythematous patches and crusting (Figure 1). The eruption compared few weeks after topical treatment with imiquimod cream used for actinic keratosis and lasted for several months without spontaneous resolution. The dermatosis did not respond to treatments with topical antibiotic and steroid. Bacteriological and mycological tests were negative. Histology was nonspecific and direct immunofluorescence was negative. On the basis of the clinical and laboratory data a diagnosis of EPDS was made. According to timing of clinical manifestations and anamnestic data, we could infer that the disease was associated with a history of actinic keratosis and androgenetic alopecia and had been triggered by a previous treatment with imiquimod. Systemic steroid therapy with prednisone 0.75 mg per kg was started and a complete resolution of the disease was observed after few weeks. Therapy was then slowly reduced and the clinical manifestations of EPDS completely resolved in about 2 months (Figure 2). The patient did not show cutaneous exacerbations of the disease and is currently in complete remission.

## 3. Discussion

EPDS is probably not an uncommon disease but, rather, an underdiagnosed one. Due to the lack of specific laboratory test and pathognomonic histologic hallmarks, its identification is always a diagnosis of exclusion. The features that should guide the physicians to the correct diagnosis are the following: chronic erosions of the scalp, scarring with pustules, elderly patients with atrophic skin and multiple actinic keratoses, negative microbiological tests, a negative direct immunofluorescence, and dermatological lesions resistant to antibiotics but responsive to corticosteroids [7]. There are many pharmacological treatments for EPDS, notably topical corticosteroids, topical tacrolimus, calcipotriol, systemic corticosteroids, isotretinoin, dapsone, and photodynamic therapy [8]. In addition to acute treatment, long-term lesions should be monitored, because there is a risk of developing squamous cell carcinoma over EPDS scar [7].

Only one case of EPDS developed after therapy with imiquimod has been reported [9]. The differentiation of EPDS from an intense local reaction to topical imiquimod is mainly based on the fact the EPDS lesions last for several months whereas skin reactions to imiquimod tend to resolve within a few weeks. Although EPDS could appear as a rare adverse effect, its incidence in our opinion is probably underestimated because the condition is generally underrecognized. It is important to consider this condition in the differential diagnosis of a patient with pustules and erosions of the scalp. A diagnosis of EPDS is fundamental for an appropriate and effective therapy because the condition, if not treated adequately, could result in scarring alopecia.

In conclusion, a diagnosis of EPDS should be taken in consideration in patients treated with imiquimod, who present a persistent erosive pustular dermatosis of the scalp.
